# 3-Bromo-Isoxazoline Derivatives Inhibit GAPDH Enzyme in PDAC Cells Triggering Autophagy and Apoptotic Cell Death

**DOI:** 10.3390/cancers14133153

**Published:** 2022-06-27

**Authors:** Raffaella Pacchiana, Nidula Mullappilly, Andrea Pinto, Stefania Bova, Stefania Forciniti, Gregorio Cullia, Elisa Dalla Pozza, Emanuela Bottani, Ilaria Decimo, Ilaria Dando, Stefano Bruno, Paola Conti, Massimo Donadelli

**Affiliations:** 1 Department of Neurosciences, Biomedicine and Movement Sciences, Section of Biochemistry, University of Verona, 37134 Verona, Italy; raffaella.pacchiana@univr.it (R.P.); nidula.mullappilly@univr.it (N.M.); stefania.forciniti@nanotec.cnr.it (S.F.); elisa.dallapozza@univr.it (E.D.P.); ilaria.dando@univr.it (I.D.); 2 Department of Food, Environmental and Nutritional Sciences (DeFENS), University of Milan, 20133 Milan, Italy; andrea.pinto@unimi.it; 3 Department of Medicine and Surgery, University of Parma, 43121 Parma, Italy; stefania.bova@unipr.it; 4 Department of Pharmaceutical Sciences, University of Milan, 20133 Milano, Italy; gregorio.cullia@gmail.com (G.C.); paola.conti@unimi.it (P.C.); 5 Department of Diagnostic and Public Health, Section of Pharmacology, University of Verona, 37134 Verona, Italy; emanuela.bottani@univr.it (E.B.); ilaria.decimo@univr.it (I.D.); 6 Food and Drug Department, University of Parma, 43124 Parma, Italy

**Keywords:** pancreas cancer, cancer metabolism, GAPDH, apoptosis, autophagy, cell death

## Abstract

**Simple Summary:**

Cancer cells largely use glycolysis to obtain both chemical energy (ATP) and metabolic intermediates for anabolic reactions, and several studies proved that the blockage of the glycolytic pathway is an efficient anticancer strategy. Glyceraldehyde-3-phosphate dehydrogenase (GAPDH), a key tetrameric glycolytic enzyme, has raised considerable attention in recent years as a potential drug target in pathological conditions in which glycolytic flux has a crucial role. In cancers, a recognized involvement of the Warburg effect, as a key mechanism for cancer-cell energetic metabolism, favouring tumour progression and invasion, has contributed to highlight human GAPDH (*h*GAPDH) as an effective drug target to specifically hit cancer cells exhibiting metabolic dependence on glycolysis, without significantly affecting normal cells. In this study, we tested the effects of 3-bromo-isoxazoline derivatives specifically designed to bind and inhibit GAPDH activity, in pancreatic ductal-adenocarcinoma cells.

**Abstract:**

A growing interest in the study of aerobic glycolysis as a key pathway for cancer-cell energetic metabolism, favouring tumour progression and invasion, has led to consider GAPDH as an effective drug target to specifically hit cancer cells. In this study, we have investigated a panel of 3-bromo-isoxazoline derivatives based on previously identified inhibitors of *Plasmodium falciparum* GAPDH (*Pf*GAPDH). The compounds are active, to a different extent, as inhibitors of human-recombinant GAPDH. They showed an antiproliferative effect on pancreatic ductal-adenocarcinoma cells (PDAC) and pancreatic-cancer stem cells (CSCs), and among them two promising compounds were selected to be tested in vivo. Interestingly, these compounds were not effective in fibroblasts. The AXP-3019 derivative was able to block PDAC-cell growth in mice xenograft without apparent toxicity. The overall results support the assumption that selective inhibition of the glycolytic pathway, by targeting GAPDH, is an effective therapy for pancreatic cancer and that 3-bromo-isoxazoline derivatives represent a new class of anti-cancer compounds targeting glycolysis.

## 1. Introduction

Pancreatic ductal adenocarcinoma (PDAC) is projected to be the second cause of cancer death in Western societies within a decade [1]. The overall 5-year survival is only 6% and in resected patients it is 25%, who only account for 15% of all cases. The extremely poor prognosis of PDAC patients and the aggressive nature of this tumour type lie in its complexity, where genetic and microenvironmental factors interact, determining delayed diagnosis, resistance to chemo-, radio-, and immuno-therapy, and uncontrolled capability to grow and metastasize. Affected by internal or external factors, PDAC cells adopt extensively distinct metabolic processes to meet their demand for growth. Rewired glucose, amino-acid, and lipid metabolism and metabolic crosstalk within the tumour microenvironment contribute to unlimited PDAC progression [2]. Altered metabolism contributes also to the modulation of apoptosis and autophagy processes and drug targets, conferring a resistant phenotype. In order to overcome resistance to therapies, a variety of experimental compounds inhibiting key metabolic pathways has emerged as a promising approach to potentiate the standard treatments for PDAC in preclinical studies [3]. These considerations constitute the rational bases to support the concept that the poor prognosis of PDAC patients might be largely improved after employing targeted therapies affecting energy metabolism.

Glycolytic flux is the central carbon metabolism process in all cells, which not only produces adenosine triphosphate (ATP) but also provides metabolic intermediates for anabolic processes that support cell proliferation. Expression levels of glucose transporters and rate-limiting enzymes regulate the rate of glycolytic flux, which is dramatically accelerated in PDAC cells even in the presence of oxygen and normal mitochondrial functionality [4]. The existence of a link between aerobic glycolysis and tumourigenesis has been known ever since Otto Warburg proposed the “Warburg effect” [5]. In normal cells, lactate generation through glycolysis is limited to anaerobic conditions, while cancer cells preferentially convert glucose into lactate through glycolysis, even under normal oxygen concentrations [6]. Lactate production and secretion is coupled with extracellular microenvironment acidification, promoting invasion and metastases and reducing drug efficacy [7]. In addition, increased glycolytic intermediates can branch out from glycolysis, providing cancer cells with crucial molecules responsible for redox homeostasis, glycosylation, and biosynthesis [8]. Moreover, the increase in glycolytic flux is a metabolic adaptation of cancer cells to ensure survival and growth in nutrient-deprived environments, which confers to the Warburg effect a key role in the metabolic reprogramming of PDAC cells.

Remarkably, levels of glyceraldehyde-3-phosphate dehydrogenase (GAPDH) were found elevated in human pancreatic adenocarcinomas compared to normal pancreas, as well as in several other cancer tissues [9]. Furthermore, we previously demonstrated that the oncogenic and mutant isoforms of p53 stabilized GAPDH cytoplasmic localization, preventing its nuclear translocation and, thus, supporting glycolysis of PDAC cells and inhibiting cancer-cell-death mechanisms mediated by nuclear GAPDH [10]. These and other observations led to an increasing interest in the study and inhibition of GAPDH to achieve a metabolic blockade of cancer cells.

Human GAPDH (*h*GAPDH) is a homo-tetramer protein composed of four identical 37-kDa subunits. In the glycolytic process, GAPDH catalyzes the phosphorylation and oxidation of glyceraldehyde-3-phosphate to 1,3-biphosphoglycerate (G-3-P) using NAD^+^ as an electron acceptor. Structural studies identified two important functional regions: (i) the glyceraldehyde-3-phosphate catalytic site, including two crucial amino acids involved in the catalytic reaction (Cys^152^ and His^179^); and (ii) the NAD^+^ binding site, a primary structure named the Rossmann fold [11].

We have previously developed a new class of covalent inhibitors of *Plasmodium falciparum* GAPDH (*Pf*GAPDH), a target for the treatment of malaria, by screening a set of 3-bromo-isoxazoline derivatives that inactivate the enzyme through a covalent, selective bond to the catalytic cysteine [12,13]. These 3-Bromo-isoxazoline derivatives were also shown to inhibit human GAPDH, for which they served as tools for the investigation of the conformational flexibility of the enzyme [14]. In the present study, we tested 3-bromo-isoxazoline derivatives selected from our previous works for antiproliferative effects in PDAC cells, to assess their potential anti-cancer activity.

## 2. Materials and Methods

### 2.1. Drugs and Chemicals

All *h*GAPDH inhibitors tested (Table 1) were designed and synthesized in the laboratory of Dr. Paola Conti at the University of Milan, Italy, following procedures previously described [12,14]. They were solubilized in absolute methanol or dimethylsulfoxide (DMSO) and stored at −80 °C until use. The autofluorescent dye monodansylcadaverine (MDC) was provided by Sigma-Aldrich (Milan, Italy) and was solubilized in methanol (10 mg/mL); gemcitabine (2′,2′-difluoro-2′-deoxycytidine; GEM) was provided by Accord Healthcare (Milan, Italy) and solubilized in sterile bi-distilled water; annexinV/FITC probe was provided by eBioscience (Thermo Fisher Scientific, Milan, Italy) and solubilized in binding buffer (10 mM HEPES/NaOH pH 7.4, 140 mM NaCl, and 2.5 mM CaCl_2_).

### 2.2. Inhibition Assays of Recombinant hGAPDH

Recombinant His-tagged *h*GAPDH was produced in Escherichia coli as already described, and covalent inhibition was assayed as reported previously [14]. Briefly, *h*GAPDH at 2 μM concentration was assayed upon incubation for 48 h at 37 °C in a buffered solution containing 10 mM TEA, 5 mM EDTA, and 10 mM sodium arseniate at pH 7.6, in the presence of the inhibitors at 10 µM concentration.

### 2.3. Cell Cultures

Primary dermal fibroblast from normal human adult (HDFa) and pancreatic adenocarcinoma cell lines (PANC-1 and MIA PaCa-2) were grown in DMEM-Glutamax medium (Thermo Fisher Scientific, Milan, Italy), supplemented with 10% fetal bovine serum (FBS) and 50 µg/mL gentamicin sulfate (all from Gibco, Thermo Fisher Scientific), and incubated at 37 °C with 5% CO_2_. All cell lines were obtained from American Type Culture Collection (ATCC; Manassas, VA, USA).

In order to generate pancreatic-cancer stem cells (CSCs), PDAC parental cell lines (PANC-1 and MIA PaCa-2) were washed twice in PBS (Thermo Fisher Scientific) and then cultured in CSC medium, DMEM/F-12 (US biological Life Sciences, Marblehead, MA, USA), and supplemented with 1 g/L glucose, B27, 1 µg/mL fungizone, 1% penicillin/streptomycin (Thermo Fisher Scientific), 5 µg/mL heparin (Sigma-Aldrich), 20 ng/mL epidermal growth factor (EGF), and 20 ng/mL fibroblast growth factor (FGF) (Peprotech, UK) at 37 °C with 5% CO_2_, as previously described by Dalla Pozza et al. [15].

### 2.4. Enzyme Assays on Cell Lysates

Cells incubated with the inhibitors at 10 µM concentration were flash-frozen and thawed before the measurements. Cell lysis was produced through three freeze-thaw cycles in 100 µL of a buffered solution containing 200 mM NaCl, 1 mM EDTA, 20 mM CHAPS, and 10% sucrose. The GAPDH activity of cell lysates was measured as reported above and normalized to the total protein content, as measured by the Bradford assay.

### 2.5. GEPIA Analysis

Gene Expression Profiling Interactive Analysis (GEPIA2, http://gepia2.cancer-pku.cn (accessed on 22 February 2022) is an open-access online tool for the interactive exploration of RNA sequencing data of 9736 tumours and 8587 normal samples from the TCGA and the Genotype-Tissue Expression (GTEx) programs [16]. In this study, GEPIA2 was used to calculate the tissue-wise expression of one gene or a multi-gene signature of glycolytic pathway in PDAC, Hexokinase 1 (HK1), Phosphoglucose Isomerase (PHI), Phosphofructokinase 1 (PFK1), Aldolase (ALDOA), Triose Phosphate Isomerase 1 (TPI1), Glyceraldehyde-3-phosphate dehydrogenase (GAPDH), Phosphoglycerate Kinase 1 (PGK1), Phosphoglycerate Mutase 1 (PGAM1), Enolase (ENO1), and Pyruvate kinase (PK).

### 2.6. Cell Proliferation Assay

Cells were seeded in 96-well plate (8 × 10^3^ cells/well for fibroblasts and 5 × 10^3^ cells/well for PDAC cells), and after 24 h they were treated with the various compounds at the concentrations and time points indicated in figure legends. At the end of the treatment period, cell proliferation for parental adherent PDAC cell lines was measured by crystal violet assay in accordance with the protocol of the manufacturer, and absorbance (A_595nm_) was measured by spectrophotometric analysis (GENios Pro, Tecan, Milan, Italy). Three independent experiments were performed for each assay condition.

Cell proliferation for PDAC CSCs growing in suspension was evaluated using resazurin assay (Immunological Science, Rome, Italy), which is based on the reduction of oxidized non-fluorescent blue resazurin to a red fluorescent dye (resorufin) by the mitochondrial respiratory chain in live cells. Resazurin solution was added in each well, and after 1 h, fluorescence was measured by Tecan GENios Pro Microplate reader (Ex_535_nm, Em_590nm_). The quantity of resorufin produced is directly proportional to the amount of living cells. Three independent experiments were performed for each assay condition.

### 2.7. Western Immunoblotting Analysis

The cells were harvested, washed in PBS, and solubilized in lysis buffer in the presence of phosphatase and protease inhibitors (50 mM Tris–HCl pH 8, 150 mM NaCl, 1% Igepal CA-630, 0.5% Na-Doc, 0.1% SDS, 1 mM Na_3_VO_4_, 1 mM NaF, 2.5 mM EDTA, 1 mM PMSF, and 1× protease inhibitor cocktail). After incubation on ice for 30 min, the lysates were centrifuged at 14,000× *g* for 10 min at 4 °C and the supernatant fractions were used for Western immunoblot analysis. The protein extracts (30 μg/lane) were resolved on a 12% SDS-polyacrylamide gel and electro-blotted onto PVDF membranes (Millipore, Milan, Italy). The membranes were blocked in 5% low-fat milk in TBST (50 mM Tris pH 7.5, 0.9% NaCl, 0.1% Tween 20) for 1 h at room temperature and probed overnight at 4 °C with rabbit monoclonal anti-glyceraldehyde 3-phosphate dehydrogenase (GAPDH) (1:1000) (Cell Signaling, Danvers, MA, USA, #5174S) and mouse monoclonal anti-vinculin (Santa Cruz Biotechnology, Heidelberg, Germany, sc-66305) antibodies. Horseradish peroxidase conjugated anti-rabbit or anti-mouse IgGs (1:3000 in blocking solution) (Upstate Biotechnology, Milan, Italy) were used as a secondary antibody. Immunodetection was carried out using chemiluminescent substrates (Amersham Pharmacia Biotech, Milan, Italy) and recorded using a HyperfilmECL (Amersham Pharmacia Biotech).

### 2.8. L-Lactate Secretion Assay

PANC-1 and MIA PaCa-2 were seeded in 96-well plate (5 × 10^3^ cells/well) and treated with 10 µM of *h*GAPDH inhibitors (AXP-3009, AXP-3018, AXP-3019) for 48 h. At the end of the treatments, culture medium has been collected, centrifuged at 1500× *g* for 10 min, and diluted six-fold in distilled H_2_O. For each sample, 25 μL has been analyzed by L-lactate assay kit (#K-LATE 07/14 Megazyme, Bray, Ireland). The amount of NADH was measured by the increase in absorbance (A_340nm_). The amount of NADH formed in the reactions is stoichiometric with the amount of L-lactate. L-lactate concentration (g/L) has been calculated in accordance with the instructions of the manufacturer: L-lactate secreted by the cells in each sample was calculated by subtracting the amount of L-lactate in the culture medium (without cells) from the amount of L-lactate in the medium from each sample. The values obtained were normalized to the number of cells in each well, measured by cell-proliferation assay.

### 2.9. Extracellular Acidification Rate (ECAR) and Oxygen Consumption Rate (OCR) Analysis

ECAR and OCR were measured in PANC-1 and MIA PaCa-2 cell lines by using a Seahorse XFe24 Extracellular Flux Analyzer (Agilent Technologies, Milan, Italy). Cells were seeded at the density of 1.5 × 10^4^ cells/well (PANC-1) or 3.0 × 10^4^ cells/well (MIA PaCa-2) in a V7 XFe24-well cell-culture microplate and treated with 10 µM of AXP-3009, AXP-3018, or AXP-3019 for 48 h, or left untreated. On the day of the assay, cells were incubated in assay medium consisting of Seahorse XF DMEM Medium (Seahorse Bioscience, cat. No. 103575-100) supplemented with 10 mM glucose, 1 mM Sodium Pyruvate, and 2 mM glutamine, pH 7.4, at 37 °C in a non-CO_2_ incubator for 1 h.

OCR and ECAR were measured at the baseline and after sequentially adding 1 mM oligomycin A (port A), 2 mM (PANC-1) or 1 mM (MIA PaCa-2) of carbonyl cyanide 4-(trifluoromethoxy) phenylhydrazone (FCCP, port B), and 0.5 mM each of Rotenone and Antimycin A (port C). The non-glycolytic acidification was subtracted from ECAR measurements by adding 50 mM of 2-deoxyglucose (port D). Raw OCR and ECAR data were normalized to the DNA content per well that was quantified with the CyQUANT Cell proliferation assay kit (Thermo Fisher Scientific, cat. No. C35007), in accordance with the instructions of the manufacturer. Data related to mitochondrial respiration were calculated as described [17]. Data related to glycolysis were calculated as follows: Basal glycolysis: ECAR_BASAL_—ECAR_2-DG_; glycolytic capacity: ECAR_OLIGO_—ECAR_2-DG_; glycolytic reserve: ECAR_OLIGO_—ECAR_BASAL_.

### 2.10. Apoptotic Assay

PANC-1 and MIA PaCa-2 cell lines were seeded in 96-well plate (5 × 10^3^ cells/well) and the day after were treated with *h*GAPDH inhibitors as indicated in figure legends and incubated for 48 h. At the end of the treatments, cells were fixed with 4% paraformaldehyde in PBS for 20 min at room temperature, washed twice with PBS, and stained with annexin V/FITC (eBioscience, Thermo Fisher Scientific) diluted in 1X binding buffer [0.1 M Hepes (pH 7.4), 1.4 M NaCl, and 25 mM CaCl_2_] for 10 min at room temperature in the dark. Cells were then washed with binding buffer, and fluorescence was measured using a multimode plate reader (Ex_485_nm and Em_535_nm) (GENios Pro, Tecan). The values were normalized on cell proliferation by crystal violet assay.

### 2.11. Autophagosome Formation Assay

In order to quantify the autophagic phenomenon, cells were treated with the various compounds and then were incubated with MDC probe, which is a selective dye for acidic vesicular organelles, such as autophagic vacuoles and autophagolysosomes. Briefly, PANC-1 and MIA PaCa-2 cell lines were seeded in 96-well plates (5 × 10^3^ cells/well) and treated with the two inhibitors (AXP-3018 and AXP-3019) at 10 μM for 48 h with or without CQ at 1 mM or 3 mM of concentration. At the end of the treatments, cells were incubated in culture medium with 50 μM MDC at 37 °C for 15 min. After incubation, cells were washed with Hanks’ buffer, and fluorescence was measured by using a multimode plate reader (Ex_340_nm and Em_535_nm) (GENios Pro, Tecan). The values were normalized on cell proliferation by crystal violet assay. Three independent experiments were performed for each assay condition. The study of the autophagic phenomenon has been carried out following the guidelines reported in Klionsky et al. [18].

### 2.12. Drug Combination Studies

PANC-1 and MIA PaCa-2 cell lines were seeded in 96-well plate (5 × 10^3^ cells/well). After 24 h cells were treated with AXP-3019 and GEM using the molar concentration ratio [AXP-3019]:[GEM] = 1:1. The combination index (CI) was calculated using the Chou-Talalay equation, which takes into account both the potency (IC_50_) and the shape of the dose-effect curve by the elaboration of CalcuSyn software (Biosoft, Cambridge, UK), as reported in Fiorini et al. [19]. The general equation for the classic isobologram is given by CI = (D)1/(Dx)1 + (D)2/(Dx)2 + [(D)1 × (D)2]/[(Dx)1 × (Dx)2], where (Dx)1 and (Dx)2 in the denominators are the doses (or concentrations) of drug 1 and drug 2 alone that give x% growth inhibition, whereas (D)1 and (D)2 in the numerators are the doses of drugs 1 and 2 in combination that also inhibited x% cell-growth inhibition (i.e., isoeffective). CI < 0.3, 0.3 < CI < 0.7, and 0.7 < CI < 1.0 values indicate strong synergism, synergism, or moderate synergism, respectively, whereas, CI = 1 and CI > 1 indicate additive and antagonism, respectively. CI/effect curves represent the CI versus the fraction (0→1) of cells killed by drug combination. The isobologram graphs using IC_25_, IC_50_, and IC_75_ values indicate the equipotent combinations of the two drugs and can be used to further analyze synergism, additivity, or antagonism. Dose-reduction index 50 (DRI_50_) represents the dose-reduction folds to obtain 50% cell growth inhibition in a combination setting, as compared to each drug alone. Throughout all experiments, we obtained a linear correlation coefficient of r > 0.90.

### 2.13. Xenograft Mice Studies

All procedures involving mice were performed in compliance with our institutional animal care guidelines (no. 30/2014-B) and following national and international directives (D.L. 4 March 2014, no. 26; directive 2010/63/EU of the European Parliament and of the Council). MIA PaCa-2 cells (1 × 10^6^ cells/mouse) were subcutaneously injected into the dorsal flank of female nude mice (Charles River Laboratories, Inc., Lecco, Italy). Twelve days after cell inoculation, 5 randomized animals were chosen for each group: control group received 200 μL of PBS vehicle solution, while the treated groups received 20 mg/kg AXP-3009 or AXP-3019 diluted in PBS, by intraperitoneal injection biweekly for 7 weeks. Body mass was recorded weekly for each animal. Tumour size was monitored weekly using a calliper, in two perpendicular dimensions parallel to the surface of the mouse. Tumour volume was calculated using the formula of V = π/6 × [(w × L)^(3/2)]. Animals were sacrificed at the end of the 7-week study period. After euthanizing the mice, the tumours were resected and weighed.

### 2.14. Statistical Analysis

Statistical analysis was performed with GraphPad Prism 5 software. Significant results were referred with a *p* value < 0.05. Values are the means of three independent experiments (±SD).

## 3. Results

### 3.1. Inhibition of Recombinant hGAPDH by 3-Bromo-Isoxazoline Derivatives

**Figure 1:**
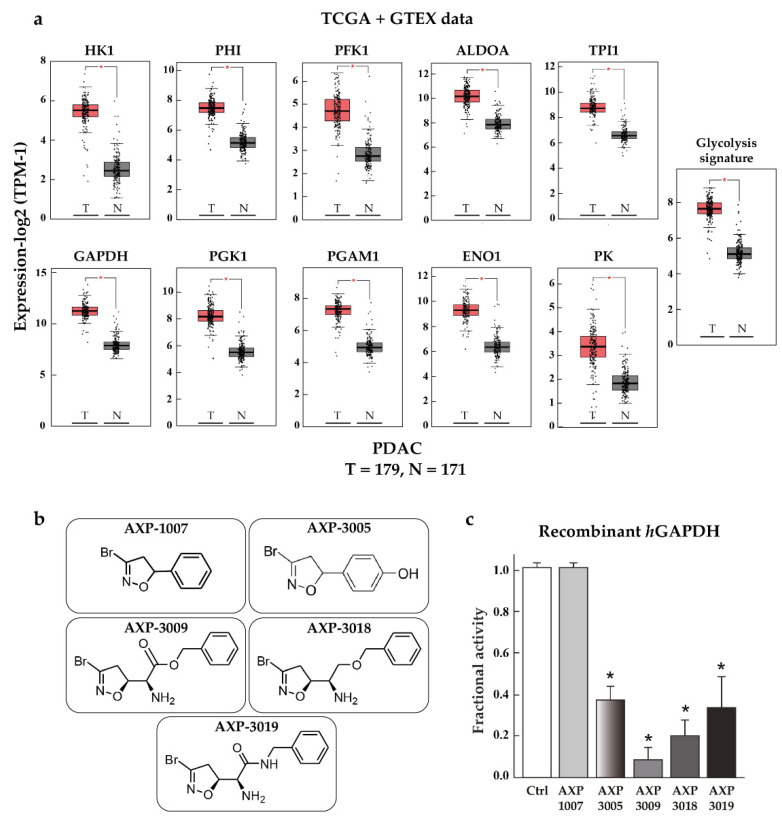
TCGA + GTEX data and *h*GAPDH activity. (**a**) Relative mRNA expression of genes regulating glycolysis pathway for normal human pancreas (N) and PDAC tissues (T), using TCGA and GTEx dataset (webtool GAPIA2). (**b**) Structures of the GAPDH inhibitors tested in this study. (**c**) Recombinant *h*GAPDH fractional activity upon incubation for 48 h at 37 °C with GAPDH inhibitors at 10 μM concentration. Statistical analysis * *p* < 0.05.

Firstly, a bioinformatic analysis using the webserver GEPIA2 was performed to determine the relative mRNA expression of the 10 glycolytic enzymes (HK1, PHI, PFK1, ALDOA, TPI1, GAPDH, PGK1, PGAM1, ENO1, PK) in PDAC tissues included in the TCGA and GTEx projects vs. the normal human pancreas (Figure 1a). The expression of all glycolytic genes showed a statistically significant upregulation in PDAC tumoural tissue compared to normal tissue. In line with this, the differential expression remained significant when the genes were combined together as a glycolytic signature. In order to therapeutically block glycolytic flux in cancer cells, we selected GAPDH as a target on the basis of our previous works, in which we tested a set of 3-bromo-isoxazoline derivatives (Figure 1b) that were designed and synthetized as inhibitors of *Pf*GAPDH [12,14]. These compounds consist of a 3-bromo-isoxazoline warhead linked to a variable recognition moiety. AXP-3005, AXP-1007, and AXP-3009 were selected as they were the best inhibitors of recombinant *Pf*GAPDH [12]. AXP-3019 was chosen as it exhibited a high anti-proliferative activity in *P. falciparum* [14], whereas AXP-3018, an analogue of both AXP-3009 and AXP-3019 in which the este/amide group was replaced with an ether, exhibited an intermediate inhibitory activity against recombinant *Pf*GAPDH [14]. In view of testing out their anti-proliferative activity in vitro, the inhibition assays were carried out on the isolated recombinant enzyme under similar conditions, i.e., at 10 µM concentration and 37 °C for 48 h. Under these conditions, AXP-1007 did not significantly inhibit recombinant *h*GAPDH (Figure 1c), although it was active at higher concentrations (data not shown). AXP-3005, AXP-3009, AXP-3018, and AXP-3019 significantly inhibited recombinant *h*GAPDH (Figure 1c).

### 3.2. Inhibition of PDAC Cell Proliferation by AXP-3009 and AXP-3019 Compounds

**Figure 2:**
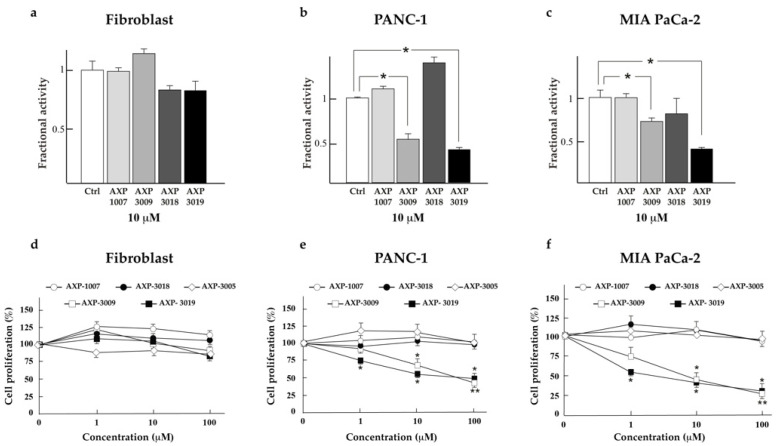
GAPDH inhibition and cell proliferation. Fibroblasts (**a**), PANC-1 (**b**) and MIA PaCa-2 (**c**) cells were left untreated or treated with GAPDH inhibitors (AXP-1007, AXP-3009, AXP-3018, AXP-3019) at 10 μM concentration for 48 h. The cells were lysed, and the GAPDH activity was measured and normalized by total protein content. The GAPDH activity is reported as the fractional activity in comparison to that of cells not treated with inhibitors. Fibroblasts (**d**), PANC-1 (**e**) and MIA PaCa-2 (**f**) were treated with GAPDH inhibitors (AXP-1007, AXP-3009, AXP-3018, AXP-3019) at three different concentrations (1 μM, 10 μM, 100 μM) for 48 h and cell proliferation was analysed using the crystal violet assay. Statistical analysis of treated vs. untreated cells * *p* < 0.05 and ** *p* < 0.001.

The various compounds were tested to effectively cross cell membranes and inhibit intracellular GAPDH. We incubated cells with the compounds, and we assayed GAPDH activity on cellular lysates. Figure 2 shows that these compounds did not affect GAPDH activity in normal fibroblasts (Figure 2a), likely for their differential membrane permeability as compared to cancer cells, while AXP-3009 and AXP-3019 significantly inhibited GAPDH in PANC-1 (Figure 2b) and MIA PaCa-2 (Figure 2c) PDAC cell lines. All compounds were then tested as inhibitors of the cellular proliferation of normal fibroblasts (Figure 2d), PANC-1 cells (Figure 2e), and MIA PaCa-2 cells (Figure 2f). In line with the results on intracellular GAPDH activity, only AXP-3009 and AXP-3019 compounds significantly inhibited PDAC cell proliferation in a concentration-dependent manner. Importantly, these compounds, even at the highest concentration tested, did not affect the proliferation of fibroblasts used as the normal control.

In order to confirm the target specificity of these enzyme inhibitors, we tested whether knocking-down the intracellular expression of GAPDH may also reduce the antiproliferative effect of these molecules. Figure S1 shows that GAPDH knock-down by a specific siRNA-GAPDH (Figure S1b) was able to neutralize the effect of AXP-3009 or AXP-3019 on the inhibition of PDAC cell proliferation (Figure S1a). This suggests the high specificity of 3-bromo-isoxazoline derivatives in inhibiting GAPDH in PDAC cells.

### 3.3. Pancreatic CSCs Are Sensitive to GAPDH Inhibition by 3-Bromo-Isoxazoline Derivatives

**Figure 3:**
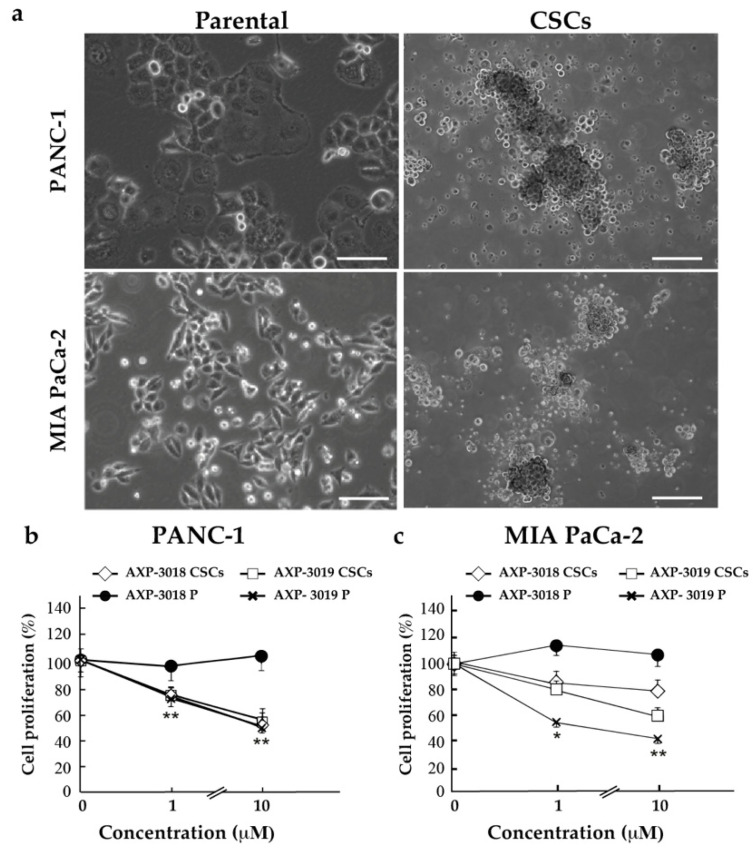
CSCs are sensitive to GAPDH inhibitors. PDAC parental (P) cells and cancer stem cells (CSCs) were treated with the *h*GAPDH inhibitors AXP-3018 or AXP-3019 at 1 μM or 10 μM. (**a**) Representative images of parental and CSCs after stemness induction. (**b**,**c**) Percentage of cell proliferation of parental (P) and CSCs after AXP-3018 or AXP-3019 treatment at the indicated conditions. The percentage of viable cells was calculated using Resazurin assay. Statistical analysis of treated vs untreated cells, * *p* < 0.05 and ** *p* < 0.001. Scale bar, 50 μM.

We also aimed to test whether pancreatic cancer stem cells (CSCs) may have a differential response to GAPDH inhibition with 3-bromo-isoxazoline derivatives as compared to the parental PDAC cells from which they were derived. In Figure 3a we show some representative images obtained by brightfield microscopy of parental and cancer stem PANC-1 and MIA PaCa-2 cells, which acquire their classic spheroidal phenotype after stemness induction, as reported in Ambrosini et al. [20].

For this purpose, we decided to use two chemically similar compounds (AXP-3018, AXP-3019) previously identified as inactive and active, respectively, on cellular proliferation in parental PDAC cells. In Figure 3b, we show PANC-1 parental and CSCs after 48 h of treatments with *h*GAPDH inhibitors AXP-3018 and AXP-3019 at two different concentrations (1 μM, 10 μM). Surprisingly, AXP-3018 acquired a strong effect in PANC-1 CSCs, while remaining ineffective in parental cells. Similarly, in MIA PaCa-2 CSCs, AXP-3018 resulted in a significant inhibition of proliferation, albeit mild, which was not evidenced in parental cells (Figure 3c). Since in vitro studies have demonstrated that AXP-3018 is a strong inhibitor of recombinant *h*GAPDH (Figure 1c), we might assume that this differential response to treatment in CSCs compared to parental cells may lie in a different composition of CSCs plasma membrane [21] and relative permeability of these compounds to these cells. On the other side, Figure 3b,c also show that the compound AXP-3019, active in parental PDAC cells, maintained its effect in both PANC-1 and MIA PaCa-2 CSCs.

### 3.4. Effects of 3-Bromo-Isoxazoline Derivatives on Glycolytic Metabolism in PDAC Cell Lines

**Figure 4:**
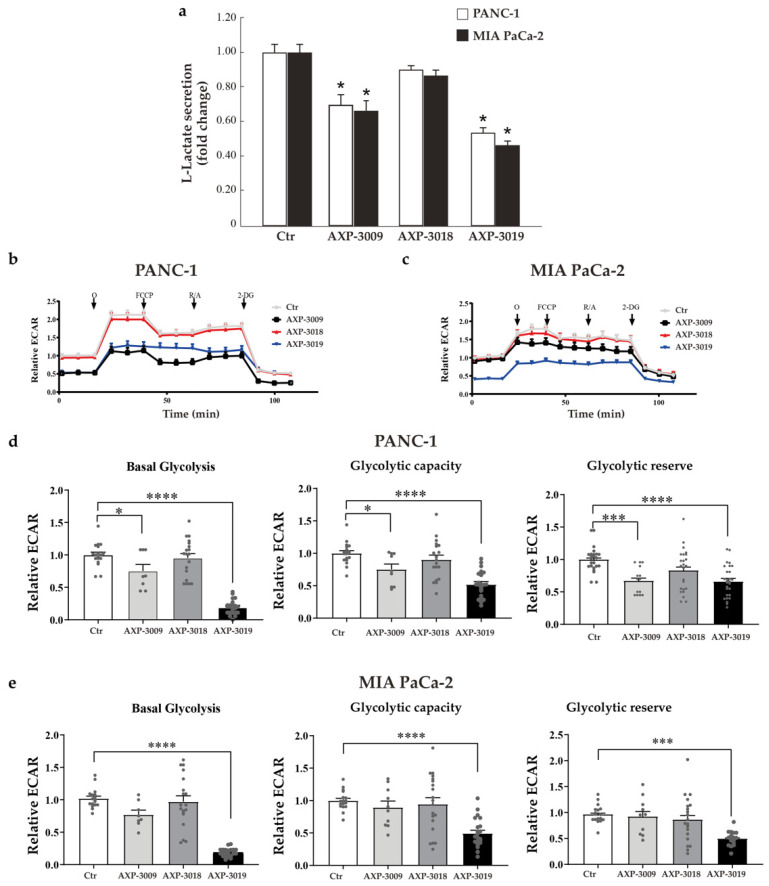
Glycolytic metabolism in PDAC cell lines. (**a**) Cells were seeded in 96-well plate and incubated with 10 μM of 3-bromo-isoxazoline derivatives for 48 h. Culture medium has been isolated and the amount of L-lactate analysed. Representative graphs of ECAR profiles of PANC-1 (**b**) and MIA PaCa-2 (**c**) treated with 10 μM of 3-bromo-isoxazoline derivatives for 48 h; O = oligomycin; FCCP = Carbonyl cyanide 4-(trifluoromethoxy)phenylhydrazone; R/A = Rotenone/Antimycin A; 2-DG = 2-Deoxy-D-glucose. Glycolytic parameters (**d**,**e**) were normalized on the total DNA content per well, calculated as detailed in Material and Methods and expressed as fold-change compared to untreated controls. Statistical analysis was performed with one-way ANOVA and Dunnett’s multiple comparisons test * *p* < 0.05,*** *p* < 0.005, **** *p*< 00001.

Since cancer cells display increased uptake of glucose and aerobic glycolysis (the Warburg effect) for their growth requirements, we analysed L-lactate secretion in PDAC cell medium after 48 h treatment with the GAPDH inhibitors AXP-3009, AXP-3018, and AXP-3019. In line with our data on GAPDH activity and cellular-proliferation inhibition, Figure 4a shows that AXP-3009 and even more AXP-3019 treatments strongly decreased L-lactate secretion in PANC-1 and MIA PaCa-2 cell lines compared to untreated cells, while AXP-3018 did not significantly decrease L-lactate secretion. This further supports a specific effect of active 3-bromo-isoxazoline derivatives on GAPDH inhibition affecting the glycolytic pathway of cancer cells.

In order to further evaluate the effects of AXP-3009, AXP-3018, and AXP-3019 in inhibiting glycolysis in PANC-1 and Mia PaCa-2 cell lines, we assessed the extracellular acidification rate (ECAR) of both cell lines in the presence of 10 mM glucose (basal glycolysis), after blocking the mitochondrial ATP production through oligomycin, a specific Fo-F1 ATP synthase inhibitor, to unravel the maximal compensatory glycolytic capacity as well as the glycolytic reserve of the cells (Figure 4b,c).

After 48 h of treatment, both the AXP-3009 and AXP-3019 drugs significantly decreased the glycolytic pathway in both cell lines, although AXP-3019 conferred the strongest impairment, while no significant differences were found after treatment with AXP-3018 (Figure 4d,e), in accordance with Figure 4a. In particular, AXP-3019 caused a strong decrease in basal glycolysis (−81% in PANC-1 and −82% in MIA PaCa-2 compared to the control, *p* < 0.0001), glycolytic capacity (−48% in PANC-1 and −51% in MIA PaCa-2, *p* < 0.0001) and glycolytic reserve (−44% in PANC-1 and −50% in MIA PaCa-2, *p* < 0.0001), indicating a severe block of the glycolytic pathway in both cell lines. The inhibitor AXP-3009 also affected the glycolytic pathway, although with varying degrees of efficiency, especially in PANC-1 cells: basal glycolysis (−25%, *p* < 0.01), glycolytic capacity (−25% *p* < 0.05), and glycolytic reserve (−33% in *p* < 0.001) (Figure 4e). These data indicate that AXP-3019 exerted the strongest inhibition of glycolysis, with consistent efficiency in both PDAC cell lines.

### 3.5. Effects of 3-Bromo-Isoxazoline Derivatives on Mitochondrial Metabolism in PDAC Cell Lines

**Figure 5:**
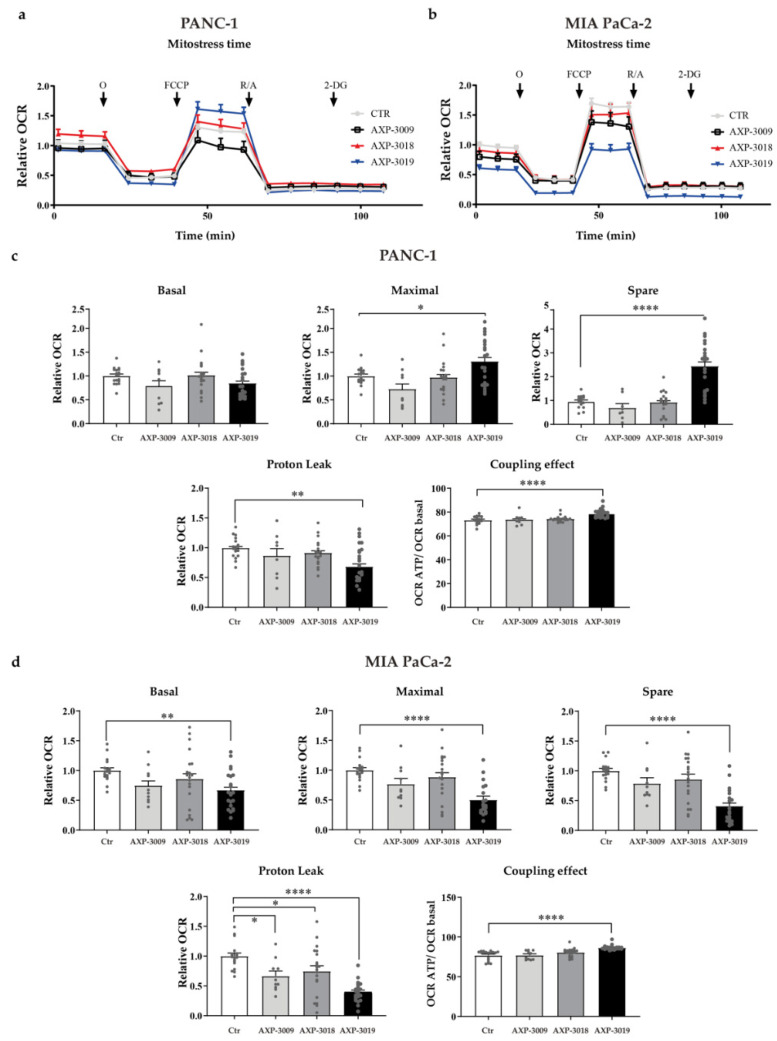
Mitochondrial metabolism in PDAC cell lines. Representative graphs of OCR profiles of PANC-1 (**a**) and MIA PaCa-2 (**b**) treated with 10 μM of 3-bromo-isoxazoline derivatives for 48 h; O = oligomycin; FCCP = Carbonyl cyanide 4-(trifluoromethoxy)phenylhydrazone; R/A = Rotenone/Antimycin A; 2-DG = 2-Deoxy-D-glucose. The parameters of mitochondrial respiration (**c**,**d**) were normalized on the total DNA content per well, calculated as detailed in Material and Methods and expressed as fold-change compared to untreated controls. Statistical analysis was performed with one-way ANOVA and Dunnett’s multiple comparisons test * *p* < 0.05, ** *p* < 0.005, **** *p*< 0.0001.

To assess whether the block of glycolysis may also impact mitochondrial metabolism, we evaluated the oxygen consumption rate (OCR) in the same experimental settings for PANC-1 (Figure 5a) and MIA PaCa-2 (Figure 5b) cell lines. Interestingly, PANC-1 and MIA PaCa-2 cell lines differentially compensate for the metabolic alterations induced by AXP-3009 or AXP-3019. In particular, PANC-1 cells treated with AXP-3019 significantly increased the depolarization-induced maximal mitochondrial oxidative phosphorylation (OXPHOS)-system rate (i.e., maximal and spare respiratory capacity, Figure 5c), and the proportion of oxygen that drives the ATP synthesis (i.e., the coupling efficiency), while reducing the proton leak through the inner-mitochondrial membrane. These data indicate that, with a severe reduction in the glycolytic-pyruvate biosynthesis, these cells can adapt their oxidative metabolism to maximize the mitochondrial respiration, possibly through alternative metabolic substrates. This could also explain the absence of significant change in basal and ATP-linked respiration, which may be compensated through alternative metabolic pathways. Accordingly, no significant modulation of mitochondrial respiration was noticed in PANC-1 cells treated with AXP-3009, which only caused mild glycolytic block.

Interestingly, the effects of the inhibitors on mitochondrial OCR were different in MIA PaCa-2 cells (Figure 5d). In particular, AXP-3019 significantly reduced the basal respiration, the spare and maximal respiratory capacity of MIA PaCa-2 cells, indicating the inability of these cell lines to cope with impaired glycolytic flux, and unraveling reduced metabolic plasticity compared to the PANC-1 cells. Again, no significant modifications were caused on OXPHOS by AXP-3009 treatment.

### 3.6. 3-Bromo-Isoxazoline Derivatives Induce Apoptosis and Autophagic Cell Death in PDAC Cell Lines

**Figure 6:**
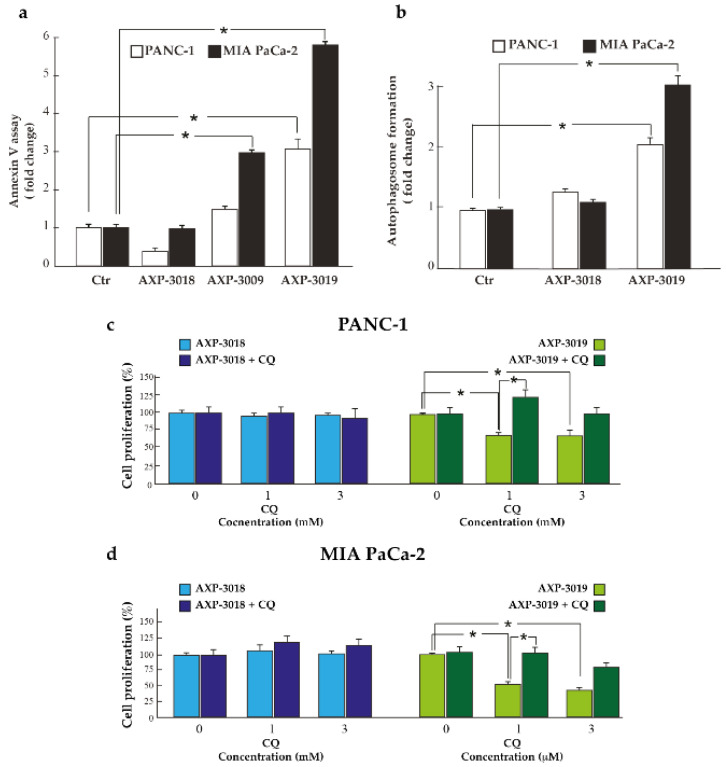
Apoptosis and autophagy events in PDAC cell lines. PDAC cell lines were seeded in 96-well plate and were treated with 10 μM of 3-bromo-isoxazoline derivatives. After 48 h, cells were fixed and stained with annexin V/FITC probe in order to analyse apoptosis (**a**) and with the fluorescent probe monodansyl-cadaverine (MDC) to quantify autophagic vesicles formation (**b**). The values were normalized on cell proliferation by crystal violet assay. In (**c**) and (**d**), the percentage of cell growth after treatment with inhibitors with or without chloroquine (CQ) is shown. Statistical analysis * *p* < 0.05.

We further investigated whether apoptosis and autophagy were involved in the inhibition of PDAC cells, after treatment with 3-bromo-isoxazoline derivatives. As shown in Figure 6a, annexin V/FITC assay revealed that PDAC cells treated with 10 μM AXP-3009 or AXP-3019 for 48 h show enhanced binding with annexin V, indicating increased externalization of phosphatidylserine and apoptotic cell death. In particular, treatment with AXP-3019 resulted in an approximately three-fold and six-fold increase in PANC-1 and MIA PaCa-2 cells, respectively, whereas AXP-3009 compound causes significant apoptotic cell death only in MIA PaCa-2 cells. In line with our previous data, AXP-3018 treatment did not induce any stimulation of annexin V binding.

To further investigate the biological events regulated by 3-bromo-isoxazoline derivatives in PDAC cells, we studied whether autophagy may be a mechanism involved in cell-proliferation inhibition. We analysed autophagosome formation by MDC assay after cellular treatment with AXP-3019 and its ineffective analogue AXP-3018. Figure 6b reveals that AXP-3019 increased MDC probe uptake (approximately two-fold in PANC-1 and three-fold in MIA PaCa-2 cell lines), indicating that GAPDH inhibition can alter the energy metabolism of cancer cells, stimulating autodigestion by autophagy. As expected, AXP-3018 failed to stimulate MDC uptake and autophagy in both cell lines tested.

In order to investigate the role of autophagy on cancer cell response to AXP-3019, we used the autophagy inhibitor chloroquine (CQ). We first checked the efficiency of CQ to repress AXP-3019-stimulated autophagy (Figure S2), without triggering cytotoxicity (data not shown), by using the experimental conditions and concentrations of CQ previously tested in these cells [19]. Importantly, we observed that CQ significantly rescued cell proliferation inhibited by AXP-3019 in both PANC-1 and MIA PaCa-2 cell lines (Figure 6c,d), indicating that the induction of autophagy constitutes a fundamental prerequisite to powerfully induce PDAC cell death by AXP-3019 treatment.

### 3.7. Gemcitabine and AXP-3019 Synergistically Inhibit PDAC Cell Proliferation

**Figure 7:**
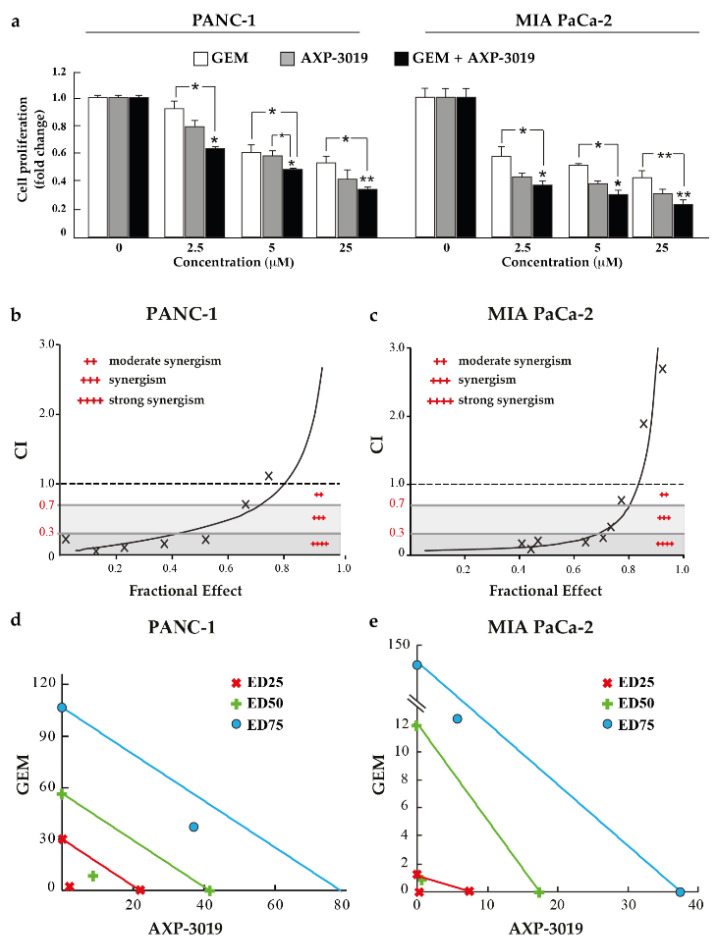
Synergistic inhibition after treatment with AXP-3019 and gemcitabine. PANC-1 and MIA PaCa-2 cell lines were seeded in 96-well plate and were treated with increasing concentrations of AXP-3019 and GEM. (**a**) Cell proliferation after treatments with AXP-3019 and GEM, alone or in combination of them (1:1 molecular ratio). (**b**,**c**) Combination index (CI) versus fractional effect (i.e., the percentage of cell growth inhibition in combined setting). (**d**,**e**) Isobologram graphs using IC_25_, IC_50_, and IC_75_ values indicate the equipotent combination of the two drugs and can be used to analyse synergism, additivity, or antagonism. Statistical analysis * *p* < 0.05; ** *p* < 0.001.

Since the Warburg effect can support aggressiveness and drug resistance of cancer cells [22], we further investigated whether the GAPDH inhibitor AXP-3019 may determine antiproliferative synergism with gemcitabine (GEM), an inhibitor of DNA polymerization used as standard treatment in PDAC patients. For this purpose, PANC-1 and MIA PaCa-2 cells were treated with increasing drug concentrations using a fixed molar ratio of the two compounds, and the results were analysed using the dedicated software Calcusyn, as described in Materials and Methods. In Figure 7a, graphs show cell proliferation of PDAC cell lines after treatment with increasing concentrations (2.5 μM, 5 μM, 25 μM) of each drug alone or their combinations at a 1:1 molar ratio. These data highlight that the combined treatment setting determined a higher antiproliferative effect than single treatments in both PDAC cell lines. Curves generated by CalcuSyn showing the combination index (CI) values vs. the fraction of cells affected/killed (fractional effect or rate of growth inhibition 0→1) by GEM and AXP-3019 in combination are illustrated in Figure 7b,c. These data demonstrate that CI values below 1 are especially distributed in the low values of the X axis, indicating that the synergistic effect mainly occurs at low drug concentrations in both PDAC cell lines. Furthermore, we report in Table 2 the CI_25_, CI_50_, and CI_75_ values of the PDAC cell lines tested and the reduction folds (dose-reduction index; DRI) of the drug concentration needed to obtain 50% cell-growth inhibition in the combination setting, as compared to each drug administered alone (DRI_50_). To deeply investigate GEM:AXP-3019 synergism, we report in Figure 7d,e the isobologram graphs using the IC_25_, IC_50_, and IC_75_ values of the PDAC cell lines tested. This reveals a strong antiproliferative synergism especially for low drug concentrations (IC_25_ and IC_50_), suggesting that this synergism might allow to decrease the concentrations of the two compounds reducing eventual side effects associated with the therapy.

### 3.8. AXP-3019 Strongly Inhibits Growth of PDAC Cells in Mice Xenografts

**Figure 8:**
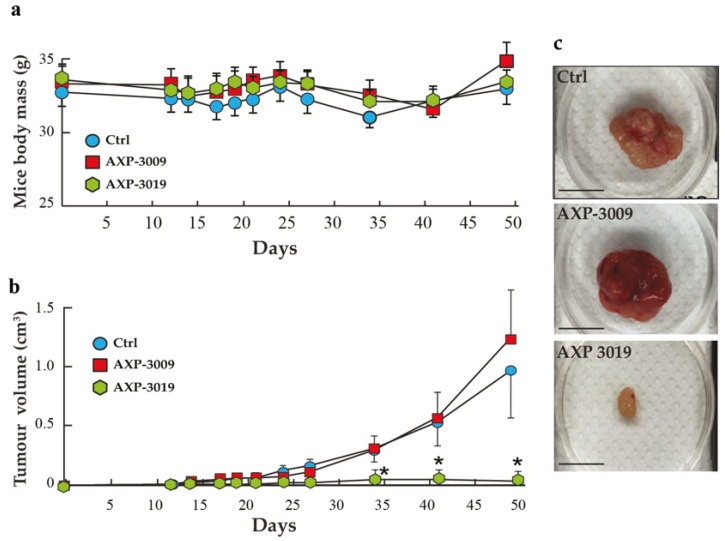
AXP-3019 strongly inhibits tumour-mass growth in mice xenografts. MIA PaCa-2 cells were subcutaneously injected into female nude mice. After 1 week, i.p. injections with PBS, AXP-3009, and AXP-3019 were administered biweekly for 30 days. Animals were sacrificed at the 50th day. (**a**) Mice body masses and (**b**) tumour volumes were measured every 2 days and values are the mean in each group. (**c**) Representative images of tumour masses grown in control, AXP-3009-, and AXP-3019-treated mice. Statistical analysis * *p* < 0.05. Scale bar, 1 cm.

In consideration of our data described before, we decided to test the effects of 3-bromo-isoxazoline derivatives AXP-3009 and AXP-3019 on the growth inhibition of the MIA PaCa-2 PDAC cell line subcutaneously xenografted in nude mice. Figure 8a shows that mice body mass did not change throughout the entire experimental period (50 days), suggesting that the treatments did not produce any apparent toxicity. Remarkably, examination of the tumour volume vs. the time curve revealed that the volume of the tumours increased considerably in the controls (vehicle) and in mice treated with AXP-3009, while they substantially did not grow in mice treated with AXP-3019, even after a long period following mice inoculation with PDAC cells (Figure 8b). Figure 8c shows representative images of tumours from untreated (vehicle), AXP-3009-treated, or AXP-3019-treated mice at the end of the experimental period. These images reproduce the results obtained by tumour volume analysis confirming the strong efficacy of AXP-3019 to inhibit PDAC cells even in vivo. On the other side, the inefficacy of AXP-3009 to inhibit tumour volume in mice is in marked contrast with cell culture results, suggesting that although this molecule can inhibit intracellular GAPDH enzyme and PDAC cell proliferation in vitro, once injected into the animals, it may be metabolized into inactive compounds. Finally, the results from AXP-3019-treated mice group are in line with cell proliferation data, supporting the hypothesis about the efficacy of this compound as a novel GAPDH inhibitor and a potential chemotherapy molecule in PDAC disease.

## 4. Discussion

In this study, we demonstrate that the 3-bromo-isoxazoline derivative AXP-3019 is able to inhibit the glycolytic enzyme GAPDH resulting in inhibition of cell proliferation and blockage of tumour-mass growth in a mouse model of human pancreatic adenocarcinoma. Interestingly, whereas several 3-bromo-isoxazoline derivatives structurally related to AXP-3019 behave as effective *h*GAPDH inhibitors, when tested against the recombinant isolated enzyme (i.e., AXP-3005, AXP-3009, AXP-3018), their actual ability to inhibit GAPDH intracellularly strongly depends on their chemical structure, which may affect their membrane permeability. Unlike other covalent modifications at the catalytic cysteine, including oxidation and S-nitrosylation [23], we have previously ruled out that the covalent binding of 3-bromo-isoxazoline derivatives affects the stability and integrity of the GAPDH tetramer [12]. Therefore, we envisage that the main mechanism of cytotoxicity resides in the inhibition of glycolysis rather than in GAPDH aggregation, a mechanism that was associated with cell death in glioblastoma cells [24].

In addition, the antiproliferative efficacy of 3-bromo-isoxazoline derivatives is variable depending on the considered cell type, as in the case of AXP-3018, which is inactive in parental cells but more active in CSCs. By further refining the selection of derivatives we have identified two molecules, AXP-3009 and AXP-3019, which are able to efficiently reduce the activity of the recombinant *h*GAPDH as well as that of the intracellular enzyme and, consequently, inhibit tumour-cell growth in vitro. Between these two molecules, only compound AXP-3019 was able to produce a significant inhibition of tumour-mass growth in vivo in a xenograft mouse model. This result may be explained by the involvement of detoxification systems, for example at the liver level, that could modify the structure of the AXP-3009 molecule rendering it inactive. We point out further relevant considerations such as the fact that compound AXP-3018, which shows no ability to inhibit the growth of parental PDAC cells, is significantly effective in inhibiting the growth of pancreatic CSCs. CSCs are an unipotent cell population present within the tumour cell mass. CSCs are known to be highly chemoresistant, and in recent years, they have gained intense interest for their tumourigenic and metastatic capacity, playing an integral role in tumour recurrence following chemotherapy [25]. Consequently, our data on the selective sensitivity of pancreatic CSCs to the AXP-3018 compound appear to be very relevant but need further investigation that may result in the synthesis of additional derivatives capable of making this feature further evident. We hypothesize that this feature is related to the differential composition of the plasma membranes of CSCs compared to parental cells of the same tissue [21]. An accumulating amount of data has indicated abnormal lipid metabolism in CSCs, and that the alteration of lipid metabolism exerts a great impact on CSCs’ properties such as the capability of self-renewal, differentiation, invasion, metastasis, and drug sensitivity or resistance [26,27]. An additional point of reflection that deserves to be discussed is that non-cancerous cells, such as human fibroblasts, are not affected at all by the treatment with any of the tested derivatives, not even with the AXP-3019 that showed the strongest antiproliferative activity in vitro (both in parental PDACs and in CSCs) and murine xenografts. Furthermore, no derivative is able to inhibit the intracellular GAPDH enzyme in fibroblasts. This event might be explained by multiple factors, including the differential composition and characteristic of plasma membranes of non-neoplastic cells, compared to both tumour cells and CSCs. In any case, it is important to note that there is a close correlation between the ability of the various derivatives to inhibit the GAPDH enzyme with that of inhibiting L-lactate secretion and inhibiting cell proliferation. This finding, together with the observation that knockdown of GAPDH expression by siRNA-GAPDH neutralizes the effect of the derivatives, strongly supports the specificity of action of these molecules on the target enzyme. All observations discussed above have been summarized in Table 3.

The block of glycolytic flux induced by the inhibition of the GAPDH enzyme determines a deep metabolic alteration in the tumour cells. In fact, it is known that cancer cells have a strong metabolic dependence on glycolysis, from which they draw not only most of the ATP produced but also numerous metabolic intermediates for biosynthetic reactions directed to the increase in tumour biomass [5]. For this purpose, it is also reported that some oncogenes render tumour cells particularly aggressive, by stimulating both glucose uptake and glycolytic flux [28]. These considerations are further strengthened by our analysis of public databases, showing increased expression of glycolytic enzymes in PDAC patients compared with the normal pancreatic counterpart. Indeed, the rationale of this study is based on this metabolic dependence, showing that PDAC cells blocked in glycolytic flux by 3-bromo-isoxazoline derivatives attempt to compensate for this deficiency with the stimulation of other catabolic pathways, as autophagy flux and OXPHOS. Autophagy is an evolutionarily ancient and highly conserved catabolic process, involving the formation of double-membraned vesicles, called autophagosomes, which engulf cellular proteins and organelles for delivery to the lysosome. Once fused with the lysosome, the contents are degraded, and the macromolecular precursors are recycled or used to fuel metabolic pathways [29]. Autophagy has opposing, context-dependent roles in cancer and interventions, to both stimulate and inhibit autophagy, which have been proposed as cancer therapies [30]. In our experimental system, we consider that stimulation of autophagic flux is an adaptation that PDAC cells adopt to compensate for treatment with glycolytic inhibitors. However, under metabolically stressful conditions such as those mentioned, excessive treatment-induced autodigestion itself becomes a cellular stressful event concomitant with the apoptotic process, as demonstrated by the fact that inhibition of autophagy by CQ prevents inhibition of proliferation by 3-bromo-isoxazoline derivatives. Autophagy and apoptosis are likely the main biological phenomena linked with the cell death induced by treatment, as also evidenced by the absence of cytostatic phenomena and alteration of cell cycle phases after treatment of PDAC cells with GAPDH inhibitors (data not shown).

In conclusion, among a panel of differently functionalized 3-bromo-isoxazoline derivatives, we identified AXP-3019 as the best molecule able to inhibit not only recombinant *h*GAPDH but also the intracellular enzyme in PDAC cells and related CSCs, without affecting GAPDH enzymatic activity in fibroblasts and their proliferation. Furthermore, AXP-3019 strongly neutralizes tumour-mass growth without apparent toxic effects in mice. Among the biological phenomena associated with the treatment, we noted the reduction in L-lactate secretion, stimulation of apoptosis, and autophagy. Finally, we demonstrated a strong antiproliferative synergy between AXP-3019 and the standard drug gemcitabine, especially at low concentrations, suggesting a possible reduction in chemotherapy doses in PDAC patients with a decrease in side effects. Finally, these data further support the connection between chemoresistance and the metabolic reprogramming of tumours, a concept that is accumulating an ever-increasing amount of scientific evidence.

## 5. Conclusions

Our data show that selected 3-bromo-isoxazoline derivatives are able to penetrate cancer cells and to bind and inhibit the GAPDH enzyme altering the cancer metabolic profile and decreasing cancer-cell growth, without affecting normal cells. Among the various analysed compounds, AXP-3019 resulted the more active inhibitor as documented by experiments in vitro and in xenograft mice. Furthermore, the capability of AXP-3019 to synergistically inhibit PDAC cell proliferation in drug-combination studies, with the standard chemotherapeutic drug gemcitabine, supports the assumption that the glycolytic enzyme GAPDH may represent an effective therapeutic target for new interventions in pancreatic-cancer cells.

## Figures and Tables

**Table 1:** The list of 3-bromo-isoxazolines derivatives tested.

Acronymous	Compound Name	Structure
AXP-1007	3-bromo-5-phenyl-4,5-dihydroisoxazole	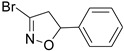
AXP-3009	(*S*)-benzyl-2-amino-2-(*S*)-3-bromo-4,5-dihydroisoxazol-5-yl-acetate	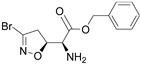
AXP-3018	(*R*)-2-(Benzyloxy)-1-((*S*)-3-bromo-4,5-dihydroisoxazol-5-yl)ethanamine	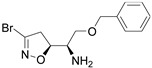
AXP-3019	(*S*)-2-Amino-*N*-benzyl-2-((*S*)-3-bromo-4,5-dihydroisoxazol-5-yl)acetamide	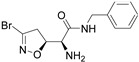
AXP-3005	4-(3-bromo-4,5-dihydroisoxazol-5-yl)phenol	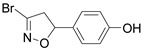

**Table 2:** Combination index (CI) and dose-reduction index (DRI) values of GEM + AXP-3019 combination on PDAC cell lines after 48 h of treatment.

Cells	Drugs	CI_25_	CI_50_	CI_75_	DRI_50_	*r*
PANC-1	GEM + AXP-3019 (1:1)	0.164	0.366	0.817	GEM 11.86 AXP-3019 8.70	0.90
MIA PaCa-2		0.052	0.111	0.433	GEM 15.61 AXP-3019 20.94	0.98

**Table 3:** Summary of the overall results.

Compound	Recombinant *h*GAPDH Activity	Intracellular GAPDH Activity			L-Lactate Secretion		Cell Proliferation				
		Fibroblast	PANC-1	MIA PaCa-2	PANC-1	MIA PaCa-2	Fibroblast	PANC-1		MIA PaCa-2	
								P	CSCs	P	CSCs
AXP-1007	**=**	**=**	**=**	**=**	***nd***	***nd***	**=**	**=**	***nd***	***=***	***nd***
AXP-3009	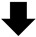	**=**	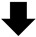	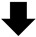	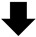	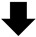	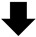	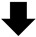	***nd***	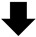	***nd***
AXP-3018	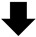	**=**	**=**	**=**	**=**	**=**	**=**	**=**	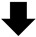	**=**	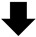
AXP-3019	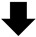	**=**	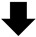	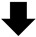	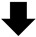	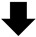	**=**	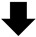	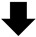	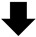	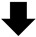

“P”, parental cells; “CSCs”, cancer stem cells; “***nd***”, not done; 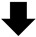 inhibition; **=** no significance difference.

## Data Availability

The data acquired from the public dataset are available at: http://gepia2.cancer-pku.cn/#index (accessed on 22 February 2022).

## Supplementary Materials

The following supporting information can be downloaded at: https://www.mdpi.com/article/10.3390/cancers14133153/s1. Figure S1: Effect of GAPDH knock-down and 3-bromo-isoxazoline-derivative AXP-3019 on cell proliferation; Figure S2: CQ reduces basal and AXP-3019-stimulated autophagy in PDAC cell lines.
