# Supplementary material for: 3-Bromo-Isoxazoline Derivatives Inhibit GAPDH Enzyme in PDAC Cells Triggering Autophagy and Apoptotic Cell Death
Source: Cancers (Basel). 2022 Jun 27;14(13):3153. doi: 10.3390/cancers14133153 (PMC9264795; doi:10.3390/cancers14133153)
Supplement: Supplementary file 1 [file cancers-14-03153-s001.zip › cancers-1774179-supplementary.pdf]

Supplementary figures

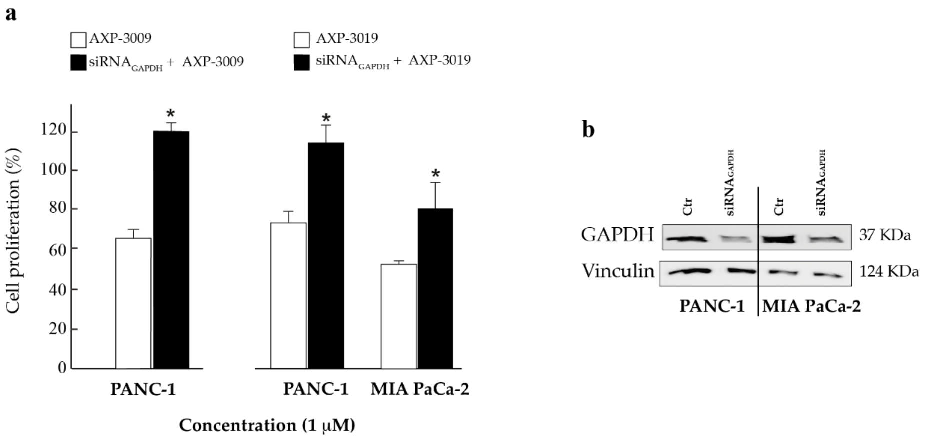

Figure S1. Effect of 3-bromo-isoxazoline derivatives in GAPDH knocking-down condition on PDAC cell proliferation. PANC-1 and MIA PaCa-2 cell lines were treated with AXP-3009 or AXP-3019 inhibitors at 1 μM for 48 h in the absence or in the presence of GAPDH silencing (siRNAGAPDH). The percentage of cell growth was calculated using crystal violet assay (**S1a**). The accompanying Western immunoblot of GAPDH and vinculin (loading control) are reported in (**S1b**). Statistical analysis * $p < 0.05$.

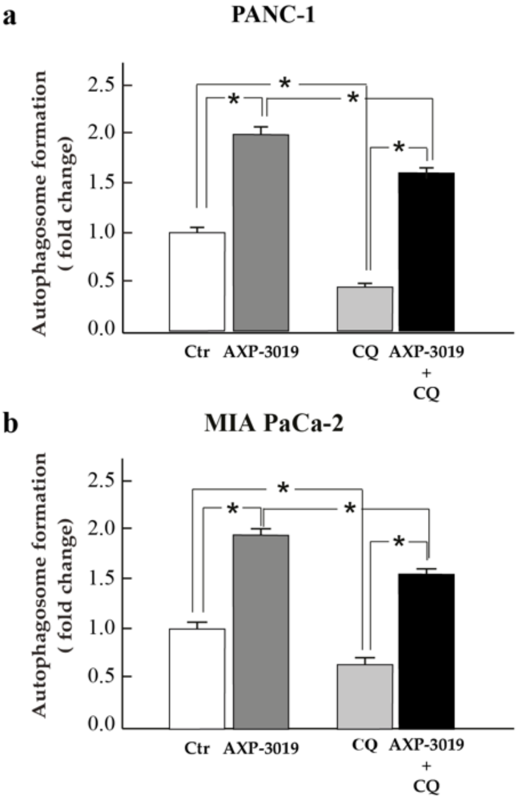

Figure S2. CQ reduces basal and AXP-3019-stimulated autophagy in PDAC cell lines. PDAC cell lines, PANC-1 (**S2a**), and MIA PaCa-2 (**S2b**) were treated with AXP-3019 alone or in combination with the autophagy inhibitor, CQ. Cells were treated with the fluorescent probe monodansyl-cadaverine (MDC) to quantify autophagic-vesicles formation. The values were normalized on cell proliferation by crystal violet assay. Statistical analysis * $p < 0.05$.
